# The Racial/Ethnic Distribution of Heat Risk–Related Land Cover in Relation to Residential Segregation

**DOI:** 10.1289/ehp.1205919

**Published:** 2013-05-14

**Authors:** Bill M. Jesdale, Rachel Morello-Frosch, Lara Cushing

**Affiliations:** 1 Department of Environmental Science, Policy and Management,; 2 School of Public Health, and; 3 Energy & Resources Group, University of California, Berkeley, Berkeley, California, USA

**Keywords:** heat risk, impervious surface, racial segregation, tree cover, urban

## Abstract

Objective: We examined the distribution of heat risk–related land cover (HRRLC) characteristics across racial/ethnic groups and degrees of residential segregation.

Methods: Block group–level tree canopy and impervious surface estimates were derived from the 2001 National Land Cover Dataset for densely populated urban areas of the United States and Puerto Rico, and linked to demographic characteristics from the 2000 Census. Racial/ethnic groups in a given block group were considered to live in HRRLC if at least half their population experienced the absence of tree canopy and at least half of the ground was covered by impervious surface (roofs, driveways, sidewalks, roads). Residential segregation was characterized for metropolitan areas in the United States and Puerto Rico using the multigroup dissimilarity index.

Results: After adjustment for ecoregion and precipitation, holding segregation level constant, non-Hispanic blacks were 52% more likely (95% CI: 37%, 69%), non-Hispanic Asians 32% more likely (95% CI: 18%, 47%), and Hispanics 21% more likely (95% CI: 8%, 35%) to live in HRRLC conditions compared with non-Hispanic whites. Within each racial/ethnic group, HRRLC conditions increased with increasing degrees of metropolitan area-level segregation. Further adjustment for home ownership and poverty did not substantially alter these results, but adjustment for population density and metropolitan area population attenuated the segregation effects, suggesting a mediating or confounding role.

Conclusions: Land cover was associated with segregation within each racial/ethnic group, which may be explained partly by the concentration of racial/ethnic minorities into densely populated neighborhoods within larger, more segregated cities. In anticipation of greater frequency and duration of extreme heat events, climate change adaptation strategies, such as planting trees in urban areas, should explicitly incorporate an environmental justice framework that addresses racial/ethnic disparities in HRRLC.

In the United States, extreme heat events are responsible for about one in five natural hazard deaths (Borden and Cutter 2008). Because of climate change, many cities are expected to become warmer [Intergovernmental Panel on Climate Change (IPCC) 2007] with “more intense, more frequent, and longer lasting” heat waves (Meehl and Tebaldi 2004). Furthermore, studies of extreme heat have shown large racial disparities in heat-related deaths (Greenberg et al. 1983; Jones et al. 1982; Kaiser et al. 2007; O’Neill et al. 2005; Schwartz 2005), although this is not universally the case (Ramlow and Kuller 1990; Weisskopf et al. 2002), and in at least one case, whites have been more affected than minority groups (Ellis et al. 1975). Land cover characteristics may contribute to these disparities (Uejio et al. 2011). Urban tree canopy is an important local mitigating factor for extreme heat (Hart and Sailor 2009; Oke et al. 1989), and impervious surfaces play a primary role in creating urban heat island effects (Oke 1982).

Urban trees provide several environmental amenities (Givoni 1991), including shade on hot days (Scott et al. 1999), reductions in wastewater loads on treatment facilities (Keim et al. 2006), and reduced air pollution (Hwang et al. 2011; Nowak 1994) and noise pollution (Samara and Tsitsoni 2011) from vehicular traffic. Research also suggests that urban trees are associated with reduced all-cause mortality after adjustment for neighborhood deprivation (Mitchell et al. 2011), and green spaces are associated with many positive health outcomes (Lee and Maheswaran 2010), including improved pregnancy outcomes (Dadvand et al. 2012). Studies in the United States have documented racial/ethnic disparities in urban tree canopy, usually in the direction of racial/ethnic minorities living in neighborhoods with lower tree coverage (Heynen et al. 2006; Landry and Chakraborty 2009; Lowry et al. 2012; Ogneva-Himmelberger et al. 2009; Perkins and Heynen 2004; Zhang et al. 2008), but some counterexamples exist (Boone et al. 2010; Troy et al. 2007). Empirical evidence does not support the notion that cultural preferences explain observed disparities in tree cover (Martin et al. 2004; Zhang et al. 2007). Most existing research on racial disparities in tree canopy has been conducted within single metropolitan areas (Boone et al. 2010; Heynen et al. 2006; Landry and Chakraborty 2009; Lowry et al. 2012; Troy et al. 2007; Zhang et al. 2008). To our knowledge, no study has examined this issue nationally or assessed the role that residential segregation plays in driving distributions of urban tree coverage among racial/ethnic groups in the United States.

Impervious surfaces, such as asphalt and concrete, contribute to urban heat islands and surface temperatures via their high heat capacity, thermal conductivity, and often low reflectance of solar radiation (Asaeda et al. 1996; Stathopoulou et al. 2009). Relative to vegetation and soil, impervious surface also reduces evapo-transporative cooling. Fine-scale, remotely sensed data has shown that impervious surfaces are important predictors of intra-urban variation in temperature (Weng and Lu 2008; Yuan and Bauer 2007; Zhang et al. 2011), and the degree of impervious surfaces generally increases with population density (Lu et al. 2006; Morton and Yuan 2009). Several authors have also found that the extent of impervious surface is greater in neighborhoods with low socioeconomic status and a high proportion of minority residents, although these studies have been limited to a single U.S. city or state (Huang et al. 2011; Li and Weng 2007; Ogneva-Himmelberger et al. 2009).

Examining disparities in land cover characteristics on a national scale could provide guidance for targeted climate change adaptation efforts to reduce future heat-related risks in U.S. urban areas. In the present study, we examined urban tree canopy and impervious surface land cover in relation to race/ethnicity and residential segregation across hundreds of urban areas in the United States and Puerto Rico, controlling for biophysical factors that may explain regional variation in tree growth, such as rainfall patterns and ecological region (e.g., desert, plains, woodlands). We also explored the potential mediating roles of population density, home ownership, and poverty.

Ultimately we sought to elucidate how social inequalities shape disparities in heat risk–related land cover (HRRLC) characteristics. Toward this goal, we used racial residential segregation as a proxy for the degree to which a metropolitan area is characterized by historical and contemporary racial inequality and discrimination (e.g., Collins and Williams 1999). Political and socioeconomic forces have led to systemic racial and ethnic segregation, with important implications for community health (Morello-Frosch 2002; Morello-Frosch and Lopez 2006). Therefore, segregation is crucial to understanding social drivers of environmental health disparities (Gee and Payne-Sturges 2004; Morello-Frosch and Jesdale 2006) and, more directly, the potentially disproportionate health burdens of climate change on communities of color (Shonkoff et al. 2011).

## Methods

Tree canopy and impervious surface land cover at the census block level were derived from the 2001 National Land Cover Dataset (NLCD) (Homer et al. 2004). Although impervious surface estimates from 2006 are available (Fry et al. 2011), no tree canopy data are included in this more recent land cover assessment, so we elected to use the 2001 data. We calculated population data at the census block level and metropolitan area segregation measures from the Summary File 1 of the 2000 census (U.S. Census Bureau 2001). Household income relative to poverty, and home ownership at the block group level came from the Summary File 3 of the 2000 Census (U.S. Census Bureau 2002). Potentially confounding regional variables were developed from Omernik ecoregions (Commission for Environmental Cooperation 1997), areas that are broadly similar in terms of ecological characteristics, such as vegetation, fauna, climate, and soils; and climate data were obtained from the National Resources Conservation Service of the U. S. Department of Agriculture (USDA 2011).

Census blocks are the finest level of detail at which population data were available. Census blocks are bounded by intersecting roads or other geographic features, and vary greatly in area and population size; within inner cities they typically correspond to a city block. Tree canopy cover at the block level was estimated by averaging the tree canopy percentage reported by the 2001 NLCD on a 30-m grid in the U.S. National Atlas Equal Area (Lambert azimuthal) projection in the 1983 North American Datum, within the land areas of blocks as assessed using 2010 Census TIGER/Line topological faces shapefiles (U.S. Census Bureau 2010), which also delineate 2000 Census boundaries. When a block included more than one land area topological face polygon, these polygons were area-weighted to the block level. A similar method was used to characterize the proportion of a block covered with impervious surfaces.

Although residents of a block are likely to live in close proximity to impervious surfaces identified in the area as a whole, the same may not be true of tree canopy within a block, especially in rural areas. To closely link both land cover measures to resident population, we considered only residents of metropolitan areas, as defined in December 2003 (U.S. Census Bureau 2004) with a 2000 population of ≥ 100,000, and further within census block groups with a population density of ≥ 2,000 persons/km^2^. Census block groups are aggregations of census blocks intended to be roughly comparable in terms of population size, typically containing between 600 and 3,000 residents. We restricted analysis to residents of owned or rented housing units for whom block-group level poverty information was available, and who identified as either Hispanic (of any race), or non-Hispanic white, black/African American, or Asian.

**Figure 1:**
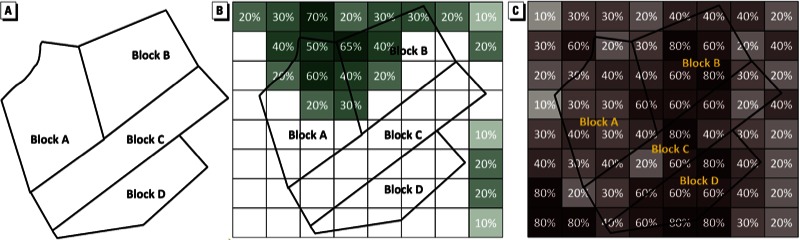
Method for assessing HRRLC characteristics. (*A*) Four blocks constituting one block group. (*B*) NLCD tree canopy overlay. (*C*) NLCD impervious surface overlay.

*Unit of analysis and assessment of HRRLC*. Each census block was classified as having either no tree canopy or some tree canopy, and as having either ≥ 50% impervious surface or < 50% impervious surface, as illustrated in Figure 1. For example, Blocks C and D would be classified as having no tree canopy (Figure 1B), and Blocks B and D would be classified as ≥ 50% impervious surface (Figure 1C). Because household poverty status was available only at the block group level, we aggregated census block–level land cover characteristics, and the weighted distribution of each of the eight subpopulations defined by race/ethnicity and housing tenure, at the census block group level for analysis:

BG land cover*_rt_* = [Σ_(_*_blocks in BG_*_)_ (block land cover × block population*_rt_*)] /BG population*_rt_,* [1]

where BG indicates block group, *r* indexes the racial/ethnic group, and *t* indexes whether they live in a rented or owner-occupied housing unit. Each subpopulation within the block group was then classified with regard to block group land cover. Specifically, if at least half of a subpopulation lived in a census block with no tree canopy, or at least half of a subpopulation lived in a census block with at least 50% impervious surface, then the subpopulation was classified at the census block group level as living with no tree canopy or with impervious surface, respectively. For example, if the census block group illustrated in Figure 1 had 50 Hispanic renters, including 10 each in Blocks A and B, none in Block C, and 30 in Block D, Hispanic renters in that block group would be classified as having no tree canopy and ≥ 50% impervious surface because > 50% of the total population in the block group lives in a census block with both of these characteristics. Thus, using this approach, Hispanics living in rented homes could have a different measure of HRRLC than Hispanics living in owner-occupied homes, depending on their relative distribution across the blocks within the block group.

*Segregation measure*. We used a multigroup dissimilarity index, D_m_ (Sakoda 1981), to characterize the unevenness of the residential distribution of the four racial/ethnic groups described above, plus a residual category consisting of all other residents, at the core-based statistical area (CBSA) level. CBSAs consist of counties or groups of counties closely linked by commuting patterns (Office of Management and Budget 2000) and we refer to them here as metropolitan areas. D_m_ ranges from 0 (i.e., no segregation, where every census block group within the CBSA has the same racial/ethnic makeup) to 1 (i.e., complete segregation, where each census block group within the CBSA consists only of a single racial/ethnic group). D_m_ describes the proportion of racial/ethnic minority populations that would need to move within the metropolitan area so that each census block group would have the same racial/ethnic makeup. Specifically,

*D_m_* = 0.5 × {[Σ*_r_*Σ*_i_* | *N_ir_* – (*N_i_N_r_*/*N*)|] /[Σ*_r_NP_r_*(1 – *P_r_*)]}, [2]

where *r* indexes each racial/ethnic group, *i* indexes the block groups, *N* is the number of residents, and *P_r_* is the proportion of residents of racial/ethnic group *r* in the entire CBSA.

We treated D_m_ as a continuous variable in the main analysis, but also performed sensitivity analyses with D_m_ modeled as a categorical variable.

*Biophysical variables*. Tree growth is dependent on ecological (or biophysical) parameters that we wanted to control for when comparing tree cover across areas of the country. Therefore, we classified each census block group according to level I ecoregions developed by Omernik (Commission for Environmental Cooperation 1997) to classify regions with similar ecological characteristics and environmental resources. We combined ecoregions that included fewer than five metropolitan areas (temperate Sierras and northwestern forested mountains, and southern semi-arid highlands and North American deserts) and assigned Hawaii and Puerto Rico to the tropical wet forests ecoregion, resulting in a variable with eight possible categories. We also considered local-area climatic variation in average annual precipitation and average precipitation during the driest month of each year for each census block group using layers developed by the USDA’s Natural Resources Conservation Service (USDA 2011). We calculated these parameters for each block group using the same projection as for the land cover characteristics.

*Analytic approach*. We used robust Poisson models to estimate prevalence ratios (Deddens and Petersen 2008) for the co-occurrence of two dichotomous heat risk-related measures: whether at least half of a subpopulation of a census block group lived in census blocks with no tree canopy reported in the NLCD and at least half of a subpopulation of a census block group lived in census blocks with at least 50% impervious surface.

We used a generalized estimating equation approach for all models to account for the fact that up to eight subpopulations might be assessed within each block group, so there were closely correlated measures for each block group. We weighted subpopulations within each block group by population, with a sum equal to the number of block groups in the analysis:

weight*_rti_* = (number of block groups × population*_rti_*)/*N*, [3]

where *r* indexes the racial/ethnic group, *t* indexes whether they live in a rented or owner-occupied housing unit, *i* indexes the block group, and *N* indicates the total size of the eligible population (81,517,417).

The first set of models we examined contained only race/ethnicity, and an interaction term between race/ethnicity and racial/ethnic residential segregation, to yield four estimates of the association between segregation and HRRLC within each racial/ethnic group:

HRRLC*_irt_* = exp(α + β*X_r_* + γ*X_r_D_mi_* + ε*_irt_*), [4]

where *i*, *r*, and *t* represent the same indices described above, *X* represents the racial/ethnic groups relative to whites, β parameterizes racial/ethnic differences relative to whites, and γ parameterizes the association of segregation with HRRLC within each racial/ethnic group.

In the second set of models, we adjusted for biophysical covariates: average annual rainfall (as six categories; < 10"/year, then in 10"/year increments, with rainfall averages > 50"/year grouped together), average rainfall in the driest month of the year (in five categories; none, < 1", 1–2", 2–3", and 3" or greater), and Omernik’s level I ecoregions (eight categories after collapsing two sparsely populated ecoregions).

In further modeling exercises, we considered variables that could alter the observed association between segregation and land cover characteristics through confounding or mediation. Home ownership and poverty have often been linked to the likelihood of having trees on both private and public lands (Heynen 2006; Heynen and Lindsey 2003; Heynen et al. 2006; Iverson and Cook 2000). These factors might theoretically be part of the causal pathway between segregation, race/ethnicity, and land cover characteristics, especially given *de jure* and *de facto* discrimination in historical and contemporary home mortgage lending that restrict where racial minority populations live (Ghent et al. 2011; Hillier 2003). We added categorical terms for housing tenure (renter vs. homeowner) and household income relative to poverty (below poverty, near poverty, or household income at least twice poverty) to the model containing the biophysical variables to assess whether associations between land cover, race/ethnicty and residential segregation seen in models controlling only for biophysical variables changed with adjustment for these variables. In sensitivity analyses, we also examined adding housing tenure terms or poverty terms separately.

In more segregated metropolitan areas, racial/ethnic minority groups tend to be clustered in densely populated neighborhoods near the central business district, and/or in “wedges” extending outwards from this central point (Berry and Kasarda 1977), whereas a ring of almost exclusively white suburban areas surrounds the metropolitan area at or near the limits of tolerable commuting distances. It is quite possible that the main effect of segregation on the distribution of land cover experienced by racial minority groups is mediated through the phenomenon of concentrating minority groups into densely populated neighborhoods. Moreover, more populous metropolitan areas tend to have a more segregated character (Iceland et al. 2002). Metropolitan area population size may precede residential segregation on the causal pathway affecting the distribution of land cover. Therefore, we examined models that included these two factors in addition to the biophysical variables.

We conducted sensitivity analyses with D_m_ modeled as a categorical versus continuous variable to assess the assumption of a linear relationship between D_m_ and the HRRLC outcome variables in the robust Poisson model, and to explore whether associations between land cover and race/ethnicity or segregation varied depending on the method we used to classify HRRLC. We also examined associations with tree canopy and impervious surface as separate components.

## Results

There were 63,436 block groups that met our eligibility criteria. These were distributed across 304 metropolitan areas, and contained 81,517,417 eligible residents, about 29% of the U.S. population in the 2000 Census. Supplemental Material, Figure S1 (http://dx.doi.org/10.1289/ehp.1205919) shows a national map of the metropolitan areas included in our analysis by level of segregation. Table 1 shows the distribution of the population across race/ethnicity, housing tenure, household income relative to poverty, and categories of D_m_. Twenty-six percent of our study population was Hispanic (of any racial identity), 19% were black, 7% Asian, and the remaining 48% were white.

**Table 1:** Proportion of urban residents living in areas with no tree canopy, high proportions of impervious surface, and both conditions, by race/ethnicity, segregation, housing tenure, and poverty.

Characteristic	Total population^*a*^ [*n* (%)]	No tree canopy (%)	≥50% impervious surface (%)	Both conditions (%)
Total population	81,517,417 (100.0)	42.1	62.2	36.5
Metro area segregation
0.13<D_m_<0.40 (97 CBSAs)	7,168,971 (8.8)	15.2	54.9	10.5
0.40≤D_m_<0.50 (105 CBSAs)	17,696,848 (21.7)	40.7	54.9	33.9
0.50≤D_m_<0.60 (78 CBSAs)	28,334,868 (34.8)	52.4	60.5	43.0
0.60≤D_m_<0.76 (24 CBSAs)	28,326,730 (34.7)	38.9	69.2	37.7
Race/ethnicity
Hispanic	21,360,877 (26.2)	56.8	72.3	49.8
Non-Hispanic
Asian	5,555,510 (6.8)	58.8	76.5	53.7
Black	15,343,325 (18.8)	34.2	61.8	31.1
White	39,257,705 (48.2)	34.4	54.0	28.6
Housing tenure
Rented housing unit	39,409,709 (48.3)	46.2	72.0	42.4
Owner occupied	42,117,708 (51.7)	37.9	52.2	30.6
Household income relative to poverty
Below poverty	14,038,788 (17.2)	46.1	68.7	41.3
Near poverty	16,283,421 (20.0)	44.8	65.4	39.4
At least twice poverty level	51,205,208 (62.8)	39.8	58.7	34.0
^***a***^Total of 81,517,417 individuals in 63,436 block groups in 304 metropolitan areas.				

Overall, 42% of the entire study population lived in block groups where at least half the population subgroup lived in blocks with no tree canopy in the NLCD, 62% lived in block groups where at least half the population subgroup lived in blocks with ≥ 50% impervious surface, and 36% lived in block groups that met both HRRLC criteria. Overall, racial/ethnic minority groups were more likely to live in areas with HRRLC than whites, particularly Hispanics and Asians. For example, 29% of whites lived in block groups with no tree canopy and mostly covered with impervious surface, as did 31% of blacks, 50% of Hispanics, and 54% of Asians. Residents of rented housing units were more likely to live in areas with both HRRLC characteristics than residents of owner-occupied housing units, and those with a household income below poverty were more likely to live in these areas than those with higher levels of household income.

Residents of metropolitan areas with a D_m_ between 0.50 and 0.60 were the most likely to have HRRLC characteristics (Table 1).

Table 2 shows modeling results for the joint occurrence of low tree canopy and high levels of impervious surface by race/ethnicity and segregation level. In the baseline models, the association between HRRLC and segregation was largest among whites (12% increased prevalence per 0.10 increase in D_m_; 95% CI: 10%, 13%), and was slightly negative among blacks. In addition, the prevalence of HRRLC for blacks, Asians, and Hispanics was about twice that of whites [e.g., a 100% increased prevalence (95% CI: 84%, 118%) for Hispanics relative to whites] after adjustment for D_m_. Racial/ethnic disparities in HRRLC remained after adjustment for Omernik ecoregion and rainfall patterns, although the magnitude of these disparities was diminished, with prevalence increased by only 21% or 52% (for Hispanics and blacks, respectively) relative to whites. In contrast, associations between segregation and HRRLC were stronger and positive for all four racial/ethnic groups (27–37% increased prevalence per 0.10-unit increase in D_m_). Further adjustment for housing tenure and household income relative to poverty had very little impact on the effect estimates; no estimates changed by ≥ 10%. However, adjustment for block group population density and metropolitan area population size in addition to Omernik ecoregion and rainfall shifted estimates for the association between HRRLC and segregation toward the null by > 10%. The disparity between Hispanics and whites increased by > 10% with this adjustment, whereas associations between black and Asian race/ethnicity and HRRLC were not substantially affected.

**Table 2:** Estimated prevalence ratios (95% CIs) for no tree canopy and at least 50% impervious surface, by race/ethnicity and multigroup dissimilarity index (D_m_).

	Model 1^*a*^	Model 2^*b*^	Model 3^*c*^	Model 4^*d*^
Whites	1.00	1.00	1.00	1.00
Per 0.10 D_m_, among whites	1.12 (1.10, 1.13)	1.34 (1.30, 1.38)	1.37 (1.33, 1.41)	1.00 (0.96, 1.04)
Blacks relative to whites	2.31 (2.09, 2.55)	1.52 (1.37, 1.69)	1.49 (1.34, 1.65)	1.55 (1.39, 1.73)
Per 0.10 D_m_, among blacks	0.98 (0.96, 1.00)	1.27 (1.23, 1.30)	1.29 (1.25, 1.32)	0.92 (0.88, 0.95)
Asians relative to whites	2.05 (1.84, 2.27)	1.32 (1.18, 1.47)	1.39 (1.24, 1.54)	1.22 (1.11, 1.35)
Per 0.10 D_m_, among Asians	1.05 (1.03, 1.07)	1.33 (1.29, 1.37)	1.34 (1.30, 1.38)	0.98 (0.94, 1.02)
Hispanics relative to whites	2.00 (1.84, 2.18)	1.21 (1.08, 1.35)	1.23 (1.10, 1.37)	1.42 (1.28, 1.58)
Per 0.10 D_m_, among Hispanics	1.06 (1.04, 1.08)	1.37 (1.32, 1.41)	1.38 (1.33, 1.42)	0.95 (0.91, 0.99)
^***a***^Model 1 contains terms for race/ethnicity, and the interaction between race/ethnicity and segregation. ^***b***^Model 1 plus level I Omernik ecoregion; average annual rainfall ( <10", 10"–19", 20"–29", 30"–39", 40"–49", ≥ 50" ); and average rainfall in driest month (0", <1", 1" to 2", 2" to 3", ≥ 3" ). ^***c***^Model 2 plus owner-occupied vs. rented housing units; household income below poverty, between poverty and 2× poverty, or at least twice poverty level. ^***d***^Model 2 plus block group population density (2,000–3,999/km^2^, 4,000–5,999/km^2^, 6,000–7,999/km^2^, 8,000–11,999/km^2^, 12,000/km^2^ and higher); CBSA population size (100,000–249,999, 250,000–499,999, 500,000–999,999, 1,000,000–2,499,999, 2,500,000–4,999,999, ≥ 5,000,000).				

*Sensitivity analyses*. Models of associations with ≥ 50% impervious surface or no tree canopy as separate outcomes [see Supplemental Material, Table S1 (http://dx.doi.org/10.1289/ehp.1205919)] suggest that segregation is more strongly associated with a lack of tree canopy cover than with impervious surface. Separate models adjusted for the biophysical variables (Omernik ecoregion and rainfall) plus either home ownership, poverty, block group population density, or metropolitan or population size (see Supplemental Material, Table S2) indicated that adjustment for both population density and metropolitan area population size decreased associations between land cover disparities and segregation levels towards the null.

Results of models in which segregation, represented by the multigroup dissimilarity index D_m_, was modeled as a categorical variable defined using “round number” cut-points (0.40, 0.50, 0.60), quartiles of the population-weighted distribution (0.467, 0.526, 0.606), and cut-points between four groups of 76 metropolitan areas (0.381, 0.4571, 0.523) were generally consistent with models of D_m_ as a continuous variable [see Supplemental Material, Table S3 (http://dx.doi.org/10.1289/ehp.1205919)]. Specifically, in most cases HRRLC increased monotonically with increasing segregation in each race/ethnicity group, though there was some heterogeneity depending on which cut-point schema is used. Using alternate cut-points to dichotomize tree canopy (i.e., < 10% or < 20% instead of no tree canopy vs. any) or impervious surface (> 70% or > 80% vs. > 50%) also did not qualitatively alter the results (see Supplemental Material, Table S4).

## Discussion

At a national scale, we found racial/ethnic disparities in HRRLC characteristics. We anticipated that these disparities might be attributable to confounding by biophysical factors that strongly influence tree growth, but found that racial disparities remained after adjustment for these factors.

Adjusting for home ownership and household poverty did not substantially alter associations between HRRLC and race/ethnicity or metropolitan area segregation levels within each racial/ethnic group. However, adjusting for block group population density and metropolitan area population size substantially attenuated effect estimates for segregation, suggesting that these variables either precede or are in the causal pathway between segregation and HRRLC characteristics. This is consistent with previous work indicating that segregation tends to concentrate racial/ethnic minority groups into densely populated neighborhoods, particularly in larger cities (Iceland et al. 2002; Lichter 1985; Massey and Denton 1989), which in turn are likely to have fewer trees and more impervious surfaces (Iverson and Cook 2000; Pozzi and Small 2001).

Given that the degree of segregation between blacks and whites is generally larger than between whites and either Asians or Hispanics (Iceland et al. 2002), we anticipated that the largest disparity in HRRLC characteristics would be between blacks and whites. At first glance, blacks and whites appeared nearly equally likely to share these adverse built environment characteristics on a national level (Table 1); the largest disparities in land cover characteristics were between whites and Asians, and between whites and Hispanics. However, after adjustment for Omernik ecoregion, precipitation patterns, and segregation (Table 2), the largest racial/ethnic disparity in HRRLC characteristics was between blacks and whites.

Living in a neighborhood with high HRRLC may not necessarily translate to greater risk of heat-related illness. Our finding of comparable prevalences of HRRLC in blacks and whites without adjustment for segregation or other factors (31% and 29%, respectively, vs. 50% for Hispanics) is not entirely consistent with evidence of higher risk of heat-related mortality among African Americans compared with whites (Basu and Ostro 2008; Greenberg et al. 1983; Kaiser et al. 2007; O’Neill et al. 2005; Schwartz 2005), and lower risk among Hispanics relative to whites (Basu and Ostro 2008; Whitman et al. 1997). However, consistent with our finding that Asians had the highest prevalence of HRRLC (54%), Asians were more likely to go to an emergency department for heat-related illnesses during California’s 2006 heat wave [risk ratio (RR) = 11.4; 95% CI: 5.5, 27, relative to a comparison period] than were whites (RR = 6.3; 95% CI: 5.4, 7.3), Hispanics (RR = 6.5; 95% CI: 5.3, 8.0), or blacks (RR = 5.3; 95% CI: 3.8, 7.4) (Knowlton et al. 2009).

Some of this inconsistency may be explained by other risk factors that are also associated with heat-related illness. Existing racial/ethnic disparities in chronic diseases that increase susceptibility to heat such as cardiovascular disease and diabetes (Bouchama et al. 2007; Schwartz 2005), differential representation in physical and outdoor occupations (Greenberg et al. 1983), unequal access to air conditioning (English et al. 2007; O’Neill et al. 2005), and social isolation (Klinenberg 2002) may explain a good deal of the observed disparate health outcomes despite relatively similar land cover characteristics between blacks and whites.

*Limitations*. The NLCD assessment of tree canopy and impervious surface was part of a project to categorize land cover across the United States; adaptation of these measures to assess local variation in heat risk introduces misclassification. An analysis of the accuracy of tree canopy and impervious surface estimates in the 2001 NLCD revealed that there was a consistent undercount of tree canopy in all regions of the country; misclassification ranged from an 11.3% overcount to a 34.7% undercount in developed areas (Nowak and Greenfield 2010). Because the NLCD used smoothing techniques to characterize 30-m pixels, areas with sparsely planted trees might be classified as having no trees whatsoever, which may tend to overestimate heat risk in densely populated neighborhoods. The degree of misclassification of impervious surface was also quite variable, from a 29.0% undercount to a 19.7% overcount across developed regions of the country. It is difficult to predict how this misclassification would bias our results.

The NLCD classification of impervious surface is intended mainly to distinguish between urbanized and non-urbanized areas, whereas the albedo of paved and roofed areas is a dominant consideration for urban heat [U.S. Environmental Protection Agency (EPA) 2011]. Harlan et al. (2006) examined eight select neighborhoods in Phoenix, Arizona, and found that areas with higher proportions of minority residents tended to have housing with darker roofs. In the absence of systematic evidence about the racial/ethnic distribution of the albedo of impervious surfaces, we are hesitant to speculate as to how accounting for albedo in addition to the presence of impervious surface would alter our observations.

Our analysis also does not account for any differences in pavement permeability, which can have a large impact on local surface temperatures (Haselbach et al. 2011), or other contributions to heat risk, including waste heat from energy consumed by cars and buildings (Rizwan et al. 2008) and the “urban canyon” effect created by tall buildings (Oke 1982).

Although the NLCD has produced more recent data on impervious surface (Fry et al. 2011), the 2001 data set remains the most recent nationally consistent assessment of tree canopy. We elected to match the impervious surface data and census data closest in time to the tree canopy data. It is possible that tree-planting efforts in metropolitan areas in the intervening years may have altered the patterns we observed. It is difficult to predict whether these tree-planting efforts would have reduced or exacerbated racial disparities in heat-risk related land cover on a national level.

The biophysical variables we used as controls may not have captured important factors that affect tree growth and are independent of the built environment. We did not account for soil composition, ground slope or aspect, proximity to riparian areas, or temperature characteristics (Lowry et al. 2012). However, unless these factors were distributed in a very different manner than the three biophysical variables we did consider, they would be unlikely to offset the dramatic differences in model results we observed after controlling for these biophysical variables.

Several analyses have attempted to predict the likely frequency of future extreme heat events (Lau and Nath 2012; Meehl and Tebaldi 2004), and some have attempted to assess the likely differential impact of these extreme heat events on specific populations, such as the elderly (Jackson et al. 2010). Our analysis did not incorporate heat-related morbidity and mortality data or climatic projections to assess potential racial/ethnic disparities in health risks from climate change; this would be an area worthy of future research.

## Conclusion

The U.S. EPA recommends both increased tree canopy and changes in roof and pavement characteristics to reduce urban heat intensity (U.S. EPA 2011). Many cities have developed plans to mitigate future heat risks, largely through adopting strategies that promote tree planting and high albedo roofs and pavements (U.S. EPA 2011). Results of this analysis highlight the idea that urban planning to mitigate future extreme heat should proactively incorporate an environmental justice perspective and address racial/ethnic disparities in land cover characteristics.

## Supplemental Material

(995 KB) PDF
