# Supplementary material for: The Racial/Ethnic Distribution of Heat Risk–Related Land Cover in Relation to Residential Segregation
Source: Environ Health Perspect. 2013 May 14;121(7):811–7. doi: 10.1289/ehp.1205919 (PMC3701995; doi:10.1289/ehp.1205919)

Supplemental Material
The Racial/Ethnic Distribution of Heat Risk-Related Land Cover in Relation to Residential Segregation

Bill M. Jesdale,[1] Rachel Morello-Frosch,[1,2] and Lara Cushing[3]

[1]Department of Environmental Science, Policy and Management, University of California, Berkeley, California, USA

[2]School of Public Health, University of California, Berkeley, California, USA

[3]Energy & Resources Group, University of California, Berkeley, California, USA

Table of Contents

2 **Supplemental Material, Table S1.** Adjusted prevalence ratios and 95% confidence limits for components of heat risk-related land cover characteristics, adjusted for Omernik ecoregion and rainfall parameters.

3 **Supplemental Material, Table S2.** Adjusted prevalence ratios and 95% confidence limits for living in an area with no tree canopy and at least 50% impervious surface, adjusted for Omernik ecoregion and rainfall parameters, plus each individual covariate as shown.

4 **Supplemental Material, Table S3.** Adjusted prevalence ratios for the joint occurrence of no tree canopy and over 50% impervious surface, relative to non-Hispanic Whites in least segregated metropolitan areas. Three methods of dividing D_m distribution into groups.

5 **Supplemental Material, Table S4.** Adjusted prevalence ratios and 95% confidence limits for various definitions of heat risk-related land cover.

6 **Supplemental Material, Figure S1.** Metro area segregation levels in the United States and Puerto Rico as characterized by the multi-group dissimilarity index (Dm).

Supplemental Material, Table S1. Adjusted prevalence ratios and 95% confidence limits for components of heat risk-related land cover characteristics, adjusted for Omernik ecoregion and rainfall parameters[a].

Parameter	no tree canopy		at least 50% impervious surface	
whites	1.00		1.00	
per 0.10 D_m, among whites	1.32	(1.29,1.36)	1.09	(1.07,1.11)
blacks relative to whites	1.35	(1.23,1.49)	0.71	(0.66,0.76)
per 0.10 D_m, among blacks	1.27	(1.23,1.30)	1.18	(1.16,1.20)
Asians relative to whites	1.05	(0.95,1.16)	1.47	(1.37,1.57)
per 0.10 D_m, among Asians	1.35	(1.31,1.40)	1.04	(1.02,1.06)
Hispanics relative to whites	1.00	(0.91,1.10)	0.96	(0.90,1.02)
per 0.10 D_m, among Hispanics	1.38	(1.34,1.42)	1.14	(1.12,1.16)

[a]Level I Omernik ecoregion; average annual rainfall, in inches (under 10", 10"-19", 20"-29", 30"-39", 40"-49", 50" and greater); average rainfall in driest month, in inches (0", under 1", 1" to 2", 2" to 3", 3" and greater.

Supplemental Material, Table S2. Adjusted prevalence ratios and 95% confidence limits for living in an area with no tree canopy and at least 50% impervious surface, adjusted for Omernik ecoregion and rainfall parameters[a], plus each individual covariate as shown.

Parameter	home ownership[b]		household income in relation to poverty[c]		block group population density[d]		CBSA population size[e]	
whites	1.00		1.00		1.00		1.00	
per 0.10 D_m, among whites	1.37	(1.33,1.40)	1.35	(1.31,1.39)	1.22	(1.19,1.26)	0.96	(0.92,0.99)
blacks relative to whites	1.46	(1.31,1.62)	1.56	(1.40,1.73)	1.54	(1.39,1.72)	1.49	(1.33,1.66)
per 0.10 D_m, among blacks	1.29	(1.25,1.33)	1.26	(1.23,1.30)	1.12	(1.08,1.15)	0.90	(0.87,0.94)
Asians relative to whites	1.39	(1.24,1.55)	1.32	(1.18,1.47)	1.21	(1.09,1.34)	1.28	(1.15,1.43)
per 0.10 D_m, among Asians	1.34	(1.29,1.38)	1.34	(1.29,1.38)	1.20	(1.16,1.24)	0.95	(0.91,0.98)
Hispanics relative to whites	1.25	(1.12,1.40)	1.18	(1.06,1.31)	1.35	(1.22,1.50)	1.39	(1.24,1.54)
per 0.10 D_m, among Hispanics	1.37	(1.33,1.42)	1.38	(1.33,1.42)	1.17	(1.13,1.21)	0.95	(0.91,0.99)

[a]Level I Omernik ecoregion; average annual rainfall (under 10", 10"-19", 20"-29", 30"-39", 40"-49", 50" and greater); average rainfall in driest month (0",under 1", 1" to 2", 2" to 3", 3" and greater).

[b]owner-occupied vs. rented housing units

[c]household income under poverty, between poverty and 2x poverty, or at least twice poverty level.

[d]block group population density (2,000-3,999/km2, 4,000-5,999/km2, 6,000-7,999/km2, 8,000-11,999/km2, 12,000/km2 and higher)

[e]CBSA (metropolitan area) population size (100,000-249,999, 250,000-499,999, 500,000-999,999, 1,000,000-2,499,999, 2,500,000-4,999,999, 5,000,000 and higher).

Supplemental Material, Table S3. Adjusted[a] prevalence ratios for the joint occurrence of no tree canopy and over 50% impervious surface, relative to non-Hispanic whites in least segregated metropolitan areas. Three methods of dividing D_m distribution into groups.

Race/ethnicity and segregation category	"round number" cut-points[b]		quartiles of population distribution[c]		76 metropolitan areas per group[d]	
non-Hispanic whites						
least	(ref)		(ref)		(ref)	
second	2.16	(1.91,2.46)	1.43	(1.38,1.49)	2.27	(1.98,2.60)
third	2.92	(2.58,3.31)	1.61	(1.50,1.73)	2.99	(2.61,3.42)
highest	4.53	(3.97,5.17)	1.41	(1.31,1.52)	2.77	(2.40,3.19)
non-Hispanic blacks						
least	1.27	(1.14,1.41)	1.23	(1.20,1.27)	1.30	(1.17,1.43)
second	2.52	(2.22,2.86)	1.63	(1.57,1.70)	2.67	(2.32,3.07)
third	2.94	(2.59,3.33)	1.74	(1.62,1.87)	3.47	(3.03,3.97)
highest	5.19	(4.54,5.93)	1.53	(1.42,1.66)	3.01	(2.61,3.47)
non-Hispanic Asians						
least	1.13	(0.98,1.29)	1.39	(1.35,1.42)	1.20	(1.04,1.39)
second	2.92	(2.57,3.31)	1.68	(1.61,1.75)	3.12	(2.72,3.58)
third	3.28	(2.90,3.71)	2.04	(1.89,2.19)	3.52	(3.08,4.03)
highest	5.85	(5.12,6.69)	1.67	(1.53,1.82)	3.49	(3.02,4.02)
Hispanics						
least	1.68	(1.50,1.88)	1.52	(1.47,1.56)	1.78	(1.58,2.02)
second	2.92	(2.58,3.32)	1.72	(1.66,1.79)	3.29	(2.87,3.78)
third	3.41	(3.02,3.86)	2.17	(2.02,2.34)	3.60	(3.15,4.12)
highest	6.79	(5.95,7.75)	2.35	(2.18,2.54)	3.95	(3.43,4.56)

[a]adjusted for level I Omernik ecoregion; average annual rainfall, in inches (under 10", 10"-19", 20"-29", 30"-39", 40"-49", 50" and greater); average rainfall in driest month, in inches (0",under 1", 1" to 2", 2" to 3", 3" and greater).

[b]"round number" cut-points for D_m: 0.13-0.399; 0.40-0.499; 0.50-0.599; 0.60-0.76.

[c]Population-weighted quartile cut-points for D_m: 0.13-0.467; 0.468-0.525; 0.526-0.605; 0.606-0.76.

[d]76 metropolitan areas per group cut-points for D_m: 0.13-0.380; 0.381-0.457; 0.4571-0.522; 0.523-0.76.

Supplemental Material, Table S4. Adjusted[a] prevalence ratios and 95% confidence limits for various definitions of heat risk-related land cover[b].

Parameter	no tree canopy and at least 50% impervious surface		no tree canopy and at least 70% impervious surface		no tree canopy and at least 80% impervious surface		under 10% tree canopy and at least 50% impervious surface		under 20% tree canopy and at least 50% impervious surface	
whites	1.00		1.00		1.00		1.00		1.00	
per 0.10 D_m among whites	1.34	(1.30,1.37)	1.57	(1.51,1.64)	1.70	(1.60,1.81)	1.12	(1.10,1.14)	1.10	(1.08,1.12)
blacks relative to whites	1.49	(1.34,1.66)	2.35	(1.95,2.82)	1.43	(1.03,1.97)	0.80	(0.75,0.86)	0.75	(0.70,0.80)
per 0.10 D_m, among blacks	1.26	(1.23,1.30)	1.40	(1.33,1.46)	1.63	(1.52,1.74)	1.19	(1.17,1.22)	1.18	(1.16,1.20)
Asians relative to whites	1.24	(1.11,1.39)	2.83	(2.26,3.54)	2.62	(1.69,4.07)	1.26	(1.17,1.35)	1.35	(1.27,1.44)
per 0.10 D_m, among Asians	1.34	(1.30,1.38)	1.39	(1.31,1.47)	1.54	(1.39,1.70)	1.11	(1.09,1.13)	1.07	(1.05,1.09)
Hispanics relative to whites	1.19	(1.06,1.32)	1.92	(1.59,2.33)	0.68	(0.50,0.94)	1.00	(0.94,1.07)	0.98	(0.92,1.04)
per 0.10 D_m, among Hispanics	1.37	(1.32,1.41)	1.53	(1.45,1.62)	2.00	(1.85,2.16)	1.17	(1.15,1.19)	1.15	(1.13,1.17)

[a]Adjusted for biophysical variables: level I Omernik ecoregion; average annual rainfall, in inches (under 10", 10"-19", 20"-29", 30"-39", 40"-49", 50" and greater); average rainfall in driest month, in inches (0",under 1", 1" to 2", 2" to 3", 3" and greater).

[b]Percent of eligible population living in heat risk-related land cover characteristics, by column: 36.3%, 17.9%, 8.9%, 56.8%, 60.2%.

Supplemental Material, Figure S1. Metro area segregation levels in the United States and Puerto Rico, as characterized by the multi-group dissimilarity index (Dm).

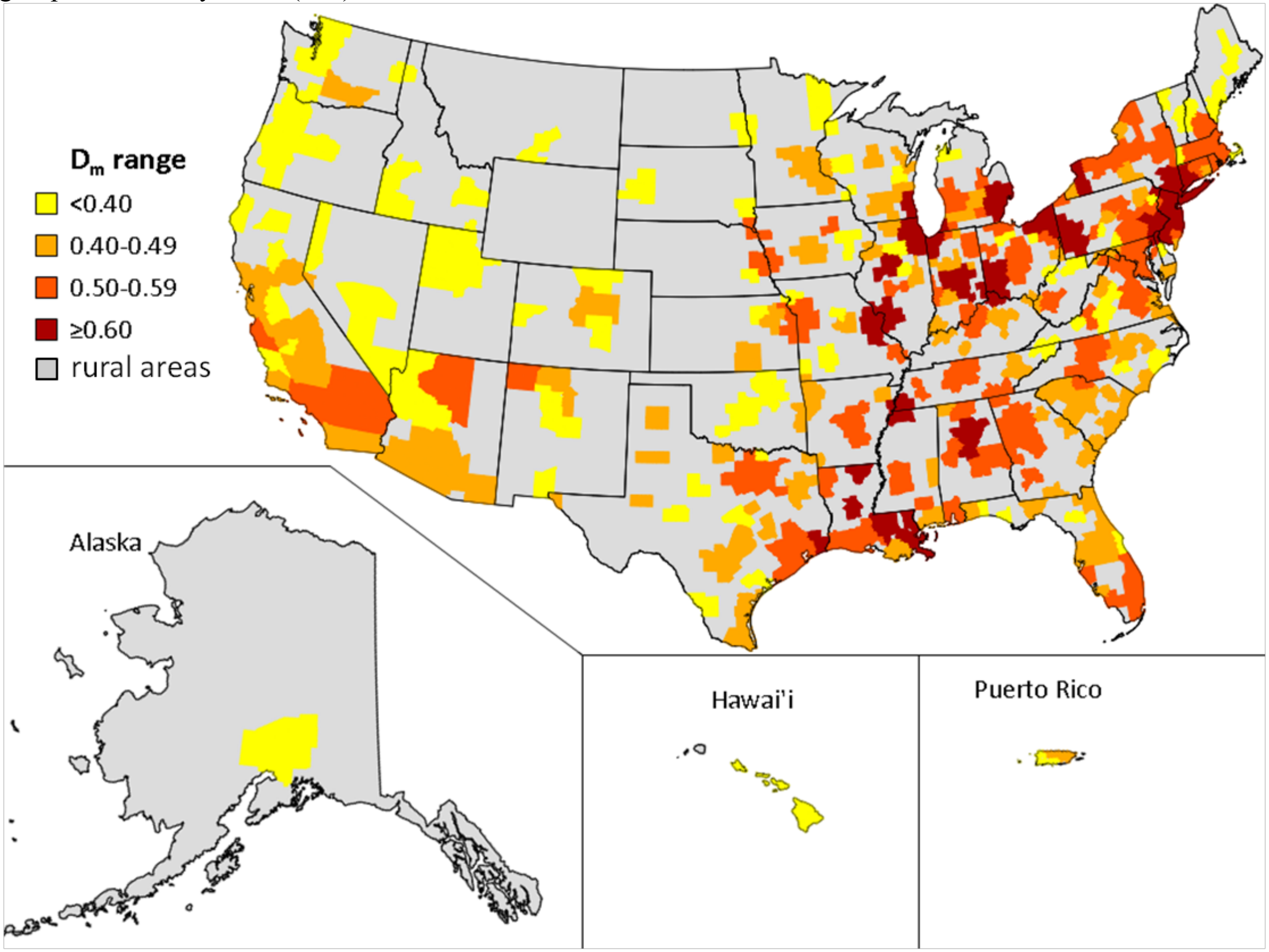

Supplement: (995 KB) PDF [file ehp.1205919.s001.pdf]
